# Figures of the Psychotherapist in Contemporary Chinese Literature

**DOI:** 10.3389/fpsyg.2022.881886

**Published:** 2022-07-26

**Authors:** Lanfang Guo, Wei Yuan

**Affiliations:** College of Foreign Languages and Cultures, Center for Comparative Literature and Transcultural Studies, Xiamen University, Xiamen, China

**Keywords:** figures of the psychotherapist, psychoanalysis in China, psychotherapy in China, reception of psychoanalysis, contemporary Chinese literature

## Abstract

Since the 1980s, psychoanalysis and psychotherapy have attracted great interest in Chinese society, and in parallel, the psychotherapist as a character has emerged in Chinese literature. Through four novels by three Chinese authors, namely, Xu Xiaobin 徐小斌, Ke Yunlu 柯云路, and Bi Shumin 毕淑敏, this article studies the evolution of the image of the psychotherapist in contemporary Chinese literature, which reflects the reception of psychoanalysis as well as the development of psychotherapy in China. In Xu Xiaobin’s novel, two psychology students attempt to cure a psychotic patient through “transference therapy” based on a complete misunderstanding of the notion; Ke Yunlu features a “life counselor” who, like typical good counselors in Chinese classical novels, simply gives advice without caring about the patients’ needs and desires; and the two novels by Bi Shumin show a significant change in the writer’s understanding of the profession and therapeutic methods, as well as the current, unregulated situation of psychotherapy in China.

## Introduction

Throughout the twentieth century and the beginning of the twenty-first century, psychoanalysis experienced a slow, staggered, and mixed reception in China. Introduced in the early 1910s, it became known to a wider audience in the 1920s, largely due to the New Culture Movement (*xin wenhua yundong* 新文化运动, 1919), which advocated for democracy and science to overcome ignorance and tyranny. Sigmund Freud’s major works, including *The Interpretation of Dreams, Introduction to Psychoanalysis*, and *New Introductory Lectures on Psychoanalysis*, were translated and published in the 1930s. During this time, influential writers who were interested in these revolutionary theories, such as Lu Xun 鲁迅, Guo Moruo 郭沫若, Yu Dafu 郁达夫, and Shi Zhecun 施蛰存, tried to apply them in their literary production. Between 1937 (the beginning of the Sino-Japanese War) and 1976 (the end of the Cultural Revolution), Freudian voices remained largely silent because of political upheavals in Chinese society.

Interest in psychoanalysis revived in China in the mid-1980s, a period marked by a “cultural fever” (*wenhua re* 文化热) or popular enthusiasm for anything related to culture, especially western modernity. In this context, the second “Freudian fever” (*Fuluoyide re* 弗洛伊德热) was born. Freud, along with Nietzsche and Sartre, was, at the time, one of the most significant western theorists to capture the imaginations of Chinese readers. In 1986 alone, dozens of publishing houses published translated works by the founder of psychoanalysis (Liu, 2008, p. 19). The studies of other renowned psychoanalysts (e.g., Carl Gustav Jung, Erich Fromm, and Karen Horney) were translated and published in the second half of the 1980s. While talk of a cooling-off of the so-called Freudian fever was common in the 1990s, the twenty-first century saw a “psycho-boom” due to various factors: “a state that is able to implement major policy changes, the disciplines of psychology and psychiatry that have striven to amplify their clout, and the market in which new commodities and business models are introduced to capitalize on the desires of consumers” (Huang, 2015, p. 14).

Since the second Freudian fever, figures of the psychotherapist have emerged in Chinese literature. Studying these figures allows us to observe the development of psychoanalysis and psychotherapy in China and to assess the traces left by psychoanalysis on Chinese literature of the period. In the present study, we examined the roles of psychotherapists in the works of three Chinese authors: Xu Xiaobin 徐小斌 (1953–), Ke Yunlu 柯云路 (1946–), and Bi Shumin 毕淑敏 (1952–), all of whom show a strong interest in exploring psychoanalysis.

Before analyzing the texts, it is necessary to delineate the designations of practitioners in the field. As noted by Zhang (2014), practitioners in China can be divided into three “camps”: those that are academy-based (*xueyuan pai* 学院派), who are university psychology professors; those that are hospital-based (*yiyuan pai* 医院派), who are mainly psychiatrists; and those that are society-based (*shehui pai* 社会派), who run private practices and who come out of the short-term certification programs (Zhang, 2014, p. 290). Huang (2018, p. 375) also referred to professors and college counselors as psychologists, which seems reasonable since the word “psychologist” can be defined as “scientist who studies and is trained in psychology” (Oxford English Dictionary). Its equivalent in Chinese is *Xinli xuejia* (心理学家). The third camp, despite lacking formal, degree-based training in psychiatry or psychology, represents the majority of Chinese psychotherapists. There are two designations for these practitioners: *Xinli zixunshi* (心理咨询师, psychological counselor) and *Xinli zhiliaoshi* (心理治疗师, psychotherapist). Initially, the terms “psychological counseling” and “psychotherapy” were used interchangeably (Huang, 2018, p. 378); however, this changed following the release of The Draft Mental Health Law in 2011, which stipulated that psychotherapists can only provide services within medical facilities. As a result, we now commonly refer to those working in private practice as “psychological counselors.” Considering that the identity of the figures we study in this article varies, we use the term “psychotherapists” in a general sense.

## Xu Xiaobin: “Transference Therapy”

Xu Xiaobin 徐小斌, writer and playwright, is renowned for the mystique aura and feminist positions that characterize her work. Her interest in psychoanalysis, which is evident in her novels, makes her writings pertinent for this project. In *Feathered Serpent* (*Yushe* 羽蛇, 1998), she emphasizes the importance of Freud in Chinese society in the 1980s: “In a certain period in the early 1980s, whether one knew Freud or not represented squarely a touchstone and a dividing line for distinguishing the elites from the masses, the nobles from the common people, the learned from the ignorant, even a clue as to whether a person was intellectual or not” (Xu, 1998a, pp. 207–208).^1^ In this novel, the treatment of trauma reveals a psychoanalytical approach. Moreover, Xu Xiaobin’s passion for psychoanalysis is not limited to Freud; in her work, Jungian terms such as *persona, anima*, and *animus* appear regularly. She is, to our knowledge, the first writer in contemporary Chinese literature to feature a “psychotherapist” as a character in fiction.

In the short story “Investigation of a psychotic” (“Dui yige jingshenbing huanzhe de diaocha” 对一个精神病患者的调查, 1985), Xu Xiaobin portrays two psychology students trying to cure a supposed psychotic patient. During their internship at a psychiatric hospital, Xie Ni 谢霓 and “I” take an interest in a young patient, Jing Huan 景焕, who is diagnosed as psychotic. Xie Ni discovers that Jing Huan is not psychotic at all but a “distorted normal person” (Xu, 1998b, p. 12). To cure Jing Huan, Xie Ni asks for “I”’s help: she wants “I” to approach Jing Huan and become her boyfriend while she plans to contact Xia Zonghua 夏宗华, Jing Huan’s ex-boyfriend, who is closely linked to Jing Huan’s illness and holds the key to the origin of her disorder. Xie Ni calls this method “transference therapy” (移情疗法) and says that it is based on the “Freudian rule (弗洛伊德定律),” a term used by “I” to refer to the attraction between the sexes. It should be noted that this takes place at the beginning of the second Freudian fever, during which the understanding of psychoanalytical theories lies, first, in the acceptance of sexual desire as a natural drive. The reintroduction of Freud’s thoughts into cultural discourse thus contributed greatly to the legitimization of love and sexuality in Chinese literature.

How did Xie Ni arrive at the idea of transference therapy? She explained this as follows when trying to persuade “I” to court Jing Huan:

You know that the heart of Jing Huan is like fire wrapped in layers of ice. Our task is to melt the ice. The remedy is love, heterosexual love above all. As I understand it, Jing Huan has never felt loved. She loves Xia Zonghua, but Xia did not give her the same love. For a long time, her life was supported by a kind of spiritual love that she imagined. This imagination inside the heart ended up later by collapsing and herself also collapsed. What I want you to do is to have her love transferred to you (Xu, 1998b, p. 22).

Xie Ni’s relating Jing Huan’s pathology to a lack of love is reasonable. Nevertheless, to address this lack, she thinks that a man’s love is needed above all, which is unconvincing. The strategy she wants to implement seems shocking because it is situated in a therapeutic context: the therapist is trying to involve the patient in a love relationship without really being in love with her. According to Xie, this simulation of love will cure Jing Huan of her psychological disorder. For Xie, this constitutes a “transference treatment.”

Admittedly, transference is an essential element of psychoanalytic therapies, and the author has understood the irreplaceable role of transference in psychoanalysis. However, neither has she grasped exactly what transference means nor can she determine how it operates to have a curative effect. Daniel Lagache explains the notion of transference in the psychoanalytic vision as follows:

Analytic transference is usually defined as the repetition, toward the analyst, of unconscious, friendly, hostile, or ambivalent emotional attitudes that the patient established in his childhood, in contact with his parents and other people around him. This definition highlights an essential aspect of transference that the patient repeats through action rather than recognizing through thought and speech. However, repetition in the therapeutic relationship does not show the full extent of transference. Transference is properly the actualization, in the psychoanalytic field, of an unconscious problem whose roots go back to childhood (Lagache, 1955–2009, p. 89).

In short, transference occurs through repetition in the current practice of the psychoanalytic situation of unconscious conflicts resulting from a child’s interactions with their parents during childhood. This means that the patient’s relationship with their parents will be projected onto their current relationship with the therapist:

The disposition to transference consists mainly in the fact that the patient’s ego activates, once again, the infantile conflict that was at the core of the neurosis. But the analytic environment is ambiguous, it encourages and disappoints; the repressed aspirations are recognized but not satisfied; the frustrations proceeding from the rule of abstinence increasingly push the patient toward trauma from the remote past and regressive forms of the transference neurosis. […] When the psychoanalyst approaches the areas where the repressed aspirations are hidden, all the defensive forces that determined the repression rise up against his efforts and manifest themselves in the defensive transference. Interpretation brings these resistances to light, in the varied forms in which they are obstinately repeated (elaboration). Progressively, the unconscious conflict, i.e., the infantile neurosis, expresses itself in a more and more identifiable form (Lagache, 1955–2009, p. 90).

To summarize, the infantile conflict in the therapeutic relationship is activated, the dissatisfaction of repressed aspirations is revealed, the appearance of the resulting defensive forces is observed, and an interpretation of these resistances is conducted. It is thus a prolonged process aimed at bringing to the conscious level, the unconscious conflict that is at the origin of the disorder. In Xu Xiaobin’s short story; however, none of these elements of transference are present. The psychotherapist’s course of treatment entirely consists of the decision to convince “I” to engage in the personal life of the patient to gain her trust, hoping that she will confide in him about the past that led to her psychic disorder.

Everything goes according to plan. Jing Huan, who, as Xie Ni pointed out, has never known tenderness, falls in love with “I,” who accompanies her and is always listening. She informs “I” about different aspects of her life, especially her family conflicts, her markedly pathological relationship with her ex-boyfriend, and the pressures of her former job as an accountant. “I” then realizes that this young patient, rather than being sick, is talented and extraordinary; her apparent disorder stems from the fact that she is different from other people and deviates from the routine and rules imposed by them. In fact, “I” is attracted to Jing Huan, despite an underlying notion that she may not be the right person for him. He tells her that he has been in a relationship with Xie Ni for years and that they have had problems because of Jing Huan’s appearance in their lives: “I try to say this in a gentle way, in a moderate tone. I don’t want to weigh down this girl’s psychic trauma. But I have to say it. It will hurt her more if I continue to hesitate” (Xu, 1998b, p. 84). This scene is cruel, not only because “I” once again makes Jing Huan experience a lover’s neglect but also because he places the blame for the disharmony in his relationship between Xie Ni and “I” on her.

So far, we see that the supposed transference therapy, instead of having a curative effect, ultimately hurts the patient. Even more shocking, the two psychology students seem not to care about professional ethics. While planning to administer their experimental treatment, they do not even consider the harm it could cause to Jing Huan.

In her essay “Recollections on Beijing University” (“Youguan beida jiyi” 有关北大记忆), Xu Xiaobin discusses her friendship with Qian Mingyi 钱铭怡, now a famous psychology professor. They have been friends since childhood. Xu states that it was she who encouraged Qian to choose psychology as a major, which was at the time a relatively neglected discipline. Interestingly, while Xu shows a great interest in psychoanalysis, her friend later becomes a leader in the development of cognitive-behavioral therapy, which, in terms of orientation, is opposite to psychoanalysis in many ways. In this essay, Xu also discusses her preparations for writing “Investigation of a psychotic.” She visited Anding Hospital 安定医院, a psychiatric hospital where Qian Mingyi was an intern. There, she met a pretty young patient who seemed rather normal to her, which made her wonder how this individual could be a psychological patient. The patient in question arguably inspired the character of Jing Huan (Xu, 2010).

When “Investigation of a psychotic” was published, readers, especially intellectuals, were starting to grasp the magnitude of Freud’s theories. Nevertheless, because access to Freud was still narrow, their understanding of psychoanalysis remained limited. We can assume that Xu Xiaobin was moderately familiar with psychotherapy and psychoanalysis, probably through her exchanges with Qian Mingyi. For example, in the short story, she mentions the Rorschach test and the Neymann–Kohlstedt test and emphasizes the role of childhood trauma in personal development. Regarding transference, however, her knowledge appears limited.

In Xu Xiaobin’s understanding of transference therapy, the therapist’s love is an important element in psychological treatment. Certainly, psychoanalysis begins with a request from the subject that is “in its essence a request for love” (Callegari, 2013, p. 127). However, love as the subject of treatment has been a sensitive issue since the beginning of psychoanalysis, notably given the intimate relations between Breuer and Anna O., and between Jung and Sabina Spielrein. In this context, Freud proposed the notion of countertransference and promoted the surgeon’s coldness for analysts:

I cannot advise my colleagues too urgently to model themselves during psychoanalytic treatment on the surgeon, who puts aside all his feelings, even his human sympathy, and concentrates his mental forces on the single aim of performing the operation as skillfully as possible. […] The justification for requiring this emotional coldness in the analyst is that it creates the most advantageous conditions for both parties: for the doctor a desirable protection for his own emotional life and for the patient the largest amount of help that we can give him today (Freud, 1912–2001, p. 115).

Like Freud, Lacan, while affirming that “the analyst is he who supports the demand of the subject” (Lacan, 1966–2001, p. 194), points out that the analyst must “respond however little to demand” (Lacan, 1966–2001, p. 209). He also specifies that “analyst’s feelings have only one possible place in the game, that of the dummy” (Lacan, 1966–2001, p. 176).

In Xu Xiaobin’s text, what “I” does in proceeding according to what he assumes is the “Freudian rule” to cure his patient with the remedy of love goes against what psychoanalysts prescribe about the patient–analyst relationship. This shows how psychoanalysis was misunderstood at the time and how dangerous such “treatment” could be.

## Ke Yunlu: Psychotherapist or “Life Counselor”

Ke Yunlu is one of the few Chinese writers to openly express an interest in psychoanalysis. A brief history of his works reveals links between his creations and psychoanalysis or psychology. Ke Yunlu first appeared on the Chinese literary stage with a novel, *Rising Star* (*Xinxing* 新星, 1984), a seminal work in the genre of “reform literature” (*gaige wenxue* 改革文学). This novel is firmly positioned in the genre of realism. The sequels, *Night and Day* (*Ye yu zhou* 夜与昼) and *Decline and Prosperity* (*Shuai yu rong* 衰与荣), released in 1986 and 1987, respectively, clearly differ from *Rising Star*, particularly in their use of the stream-of-consciousness technique. This technique is closely connected to psychoanalysis, and, notably, the years 1985 and 1986 represent the most significant dates of the second Freudian fever in China. In *Decline and Prosperity* alone, the author mentions Freud 11 times. In *Night and Day* and *Decline and Prosperity*, several typical Freudian themes are evoked and discussed, including sexual impulses and their civilizational repression, dreams, and fantasies.

In 2000, Ke Yunlu published *Ignorance* (*Mengmei* 矇昧), a long story about a boy and his teacher during the cultural revolution. The relationship between these two characters has Oedipal undertones. Then, 2 years later, his psychological work *Personalities in Fairy Tales* (*Tonghua renge* 童话人格) was published, in which he attempts to psychoanalyze certain Chinese myths and Western fairy tales. In 2007, *The Anxiety Patient* (*Jiaolü zheng huanzhe* 焦虑症患者) reported a clinical case on anxiety disorders. We can ascertain from one of his interviews that he probably performed psychological consultations for his relatives (Ke, 2011).

*Decline and Prosperity* is a panoramic description of China’s reform and changing society in the 1980s. The many new developments of the time constitute important themes in the novel. For example, the author describes salons, organized by celebrities, where young people discuss everything from the literature and history to news, politics, and the future of the country. The emergence of new professions in Chinese society, such as those of actors, directors, and psychotherapists, is also evidenced in the work.

One of the characters in the novel, Chen Xiaoshi 陈晓时, supported by the National Women’s Federation and the Chinese Academy of Social Sciences, opens a Life Counseling Institute (人生咨询所), which can be understood as a psychological counseling institute, although the author does not use this term explicitly. The service the institute offers is a kind of talking therapy. Clients are mainly experiencing family or relationship issues. The staff includes a sociologist and an editor of an academic journal. They insist on confidentiality in their work and meet occasionally to discuss difficult cases. These counselors are influenced by psychoanalysis. One day, discussing with a client they diagnose as neurotic, they have differing views. One counselor blames Chen Xiaoshi for always trying to manipulate others, telling Chen: “I hope you can look back on your childhood, entrust the materials of your psyche to us, and do a self-analysis” (Ke, 2008, p. 610). This criticism leads to an analysis of Chen by his colleagues, which reveals obsessive symptoms. In fact, Chen is aware of his psychological problems and has already started self-analysis. One day, reflecting on his vertigo, he suspects that his symptoms emerged from a quarrel he had with his ex-girlfriend during which he threatened to jump off the building. He asks himself, “According to the psychology analysis, what childhood trauma did this quarrel activate?” (Ke, 2008, p. 104).

It seems credible that the novel’s counselors are familiar with psychoanalytical theories. Because other approaches are not mentioned in the text, we assume that their work is based on the analytic method. Nevertheless, analytic discourse is almost absent from their consultations. Several of Chen Xiaoshi’s consultation sessions feature in the novel, but the only moment of psychoanalytical practice takes place when Chen receives a client, a student having an affair with her professor, a married man in his forties. The student is shy, and so Chen makes assumptions and waits for her confirmation to find out where the relationship presently stands. Chen then asks, “Do you want to hear my assessment?” (Ke, 2008, p. 365). This indicates the beginning of the essential part of the so-called consultation or, more precisely, the dissemination of advice. On receiving the student’s assent, Chen says, “You are Freudianized. Everyone is” (Ke, 2008, p. 365).

It may be difficult for the student and for readers to understand this phrase; thus, a monolog by Chen Xiaoshi follows:

She, a Chinese student in 1982, does not know Freud. He laughs to himself, “Even if she knows Freud, she won’t know what I’m referring to with this name.” It’s a specific term when he jokes with his wife. (When he sees two high school students, a boy and a girl, bent over a bicycle, looking for topics to continue their conversations, he says to his wife, “Look, these two students are very Freudian.” When they hear a little girl saying that she loves Daddy the most, they look at each other and smile: always, it’s Freud.) (Ke, 2008, p. 365).

In this context, Freud is synonymous with sexuality. In fact, this is how the theorist was received when he was reintroduced in China in the 1980s. With the statement “You are Freudianized. Everyone is,” Chen Xiaoshi tells the girl that if she expresses sympathy toward her teacher, it is understandable because such feelings are natural between the sexes. He talks to her in this manner in the hope that she will not feel guilty. Chen later states that what her teacher did already deviates from “Freud,” because, in his opinion, the teacher is taking advantage of his position to manipulate the student. We cannot contemplate Chen’s reasons for this conclusion, because the girl’s statement is rather short and simple. He ends the consultation by giving advice, which comprises seven specific tips – a list of do’s and don’ts – written on a card to help the student to extricate herself from the relationship with the teacher without offending him.

During the consultation, except for a brief description of her troubles, the student is mostly silent. When Chen asks her questions, she answers with “yes” or “no,” or by nodding. It is always the counselor who makes long speeches, which creates the impression of a puppet and manipulator. The counselor is placed on the position of the “subject [who is] supposed to know,” in Lacan’s words. Chen sees himself as knowledgeable and being able to guide the clients through a psychological labyrinth. He gives advice firmly, as if it were absolute truth. Indeed, the counselors of the Life Consulting Institute consider themselves the agents of enlightenment. They want to establish a newspaper named *Life Counseling* (人生咨询报), which will be a periodical of enlightenment aiming to promote new ideas. This reveals the purpose of their consultation to develop people’s awareness.

As mentioned above, the counselors at the institute possibly consider psychoanalysis a functional method of treatment. However, what they do in practice – lecturing clients to awaken them and telling them exactly what to do and not do – is far removed from the aims of psychoanalysis. Jean-Pierre Cléro summarizes the aim of analysis as follows:

to make the analysand understand that he is the one who knows, and the analyst, who is supposed to know, just needs to lead the analysand to speak out, from his own desire, the words that he himself would have said, the words “in which he recognizes the law of his being.” Nothing is triumphant about the end of the analysis, either on the side of the analysand or of the analyst, who hides himself, and whose desire has been in the trash bin (Cléro, 2012, p. 83).

However, in the example above, the analysand’s desire is completely drowned in that of the analyst.

Chen Xiaoshi’s consultation can be summarized as an explanation and advice. His mode of advice (e.g., writing down keywords on a small card) is reminiscent of the Chinese expression *Jinnang miaoji* 锦囊妙计 (“good stratagems contained in a brocade bag”). In Chinese classical novels, good counselors – often wise and inventive – would write strategies for defeating enemies on a small piece of paper and put it in a brocade bag. The way Chen Xiaoshi is portrayed implies that the counselor is wise and the advice he gives is a magic remedy for all problems. Thus, even though a new profession is created, whether it is called life counselor or psychotherapist, the author remains in the classic logic of wise men giving the advice to help the people to overcome the difficulties.

However, giving advice is not the objective of counseling. As Corey notes, “The counseling task is to assist individuals in finding answers that are most congruent with their own values. It is not beneficial to provide advice or to give clients your [the counselor’s] answers to their questions about life.” (Corey, 1977–2017, p. 23). What counselors are supposed to do is to help clients find their own solutions and acknowledge their own power to act, instead of imposing their opinions or values onto their clients. As for psychoanalytic psychotherapy, on which the counselors in the novel seem to rely, the avoidance of advice giving is also considered one of the basic boundary issues (Curtis and Hirsch, 2011, p. 87).

The counselors in the fiction, and probably the author himself – at least at the time of writing the novel – may have knowledge of Freud’s thoughts, but at the clinical level, they are unable to really understand the essential concerns. It should be noted that, like Xu Xiaobin’s short story, the novel was published around the time psychoanalysis was being reintroduced into the cultural discourse of China. However, even today, the diffusion of psychoanalytical theory in the Chinese context is, as Rainier Lanselle observes, “very rarely a transmission of the clinic, but rather a diffusion of the “body of doctrine”” (Lanselle, 2007, p. 136).

## Bi Shumin: From Psychotherapist-God to Apprentice Psychotherapist

In the Chinese literary community, Bi Shumin is a remarkable figure, a doctor, writer, and psychotherapist simultaneously. After working as a doctor for 20 years, from 1987, she devoted herself to writing. Then, 11 years later, she enrolled in a master’s degree in psychology at Beijing Normal University and later pursued doctoral studies in the same field (although she did not complete the PhD). In the meantime, she started working as a psychotherapist and was even dubbed the “number one Chinese psychological counselor” (中国心理咨询第一人). This does not make her the best psychological counselor in China. In our opinion, the main reason she was given this title is her reputation as a famous writer, which secured for her a large number of patients. According to an interview at the end of 2004, Bi Shumin had not yet seen patients who had made an appointment with her in March of that year (Bi, 2004).

Because of her heavy workload, Bi Shumin closed her practice 3 years later to devote herself solely to writing, mainly on topics related to psychology, in the form of essays and novels. Indeed, Bi Shumin’s literary creations are often linked to her professions – to medicine in the 1990s and to psychology in the 2000s. In 2003 and 2008, she published two novels featuring psychotherapists as their protagonists, *Saving Breasts* (*Zhengjiu rufang* 拯救乳房) and *The Woman Psychotherapist* (*Nü xinlishi* 女心理师).

The protagonist of *Saving Breasts*, Cheng Yuanqing 程远青, is a young doctor of psychology who has just returned from the United States. She decides to conduct group therapy to treat people with breast cancer because she associates the disease with psychological problems. At the novel’s end, the participants seem to be “cured” (psychically), and this makes the group a harmonious community.

Cheng Yuanqing is described as capable, and she is aware of each patient’s problem and knows exactly how to help them. Her therapy is effective, and her patients venerate her for this near-omniscient ability. An exchange between Cheng Yuanqing and one of her patients, Lu Lu 鹿路, highlights this outpouring of admiration. Lu Lu was an orphan who fell in love with the third son of her adoptive family at a young age. While she was in high school, her adoptive brother was stricken with a rare disease and required expensive treatment. To help him recover, Lu Lu quit her studies and became a prostitute. The conversation between Cheng Yuanqing and Lu Lu proceeds as follows:

Chen Yuanqing stroked Lu Lu’s hands and said, “Lu Lu, I know you are thinking that one day, when you have earned enough money, when you have helped your brother get a new kidney so that he can live like a normal person, you will never do this job again. You’ll go far away with your brother, to a place where no one will know your past. You’ll marry your brother, and you’ll take care of him for life …”

Hearing this, Lu Lu suddenly stood up, opened her eyes wide with terror and said, “Prof. Cheng, are you a deity or a ghost? I didn’t tell anyone; how did you find out? Are you sent by Heaven to save me?” She stepped back, trembling, and said to herself, “You … are you my mother? No, you can’t be. My mother is a poor, unhappy woman, how could she have such rich knowledges as you? You’re not her age either. But how could you know all this? Would you have acquired, abroad, a device to detect the secrets of others? If not, you must be inhabited by a deity?” (Bi, 2013, p. 187).

The psychotherapist certainly captures the contents of Lu Lu’s fantasy with a degree of accuracy. However, in our opinion, anyone paying attention to Lu Lu’s past, and who is sensitive to her situation, could guess her inner thoughts. Lu Lu’s reactions in this context seem melodramatic and far-fetched. Of course, as a pariah who has been mistreated and considers herself worthless, Lu Lu is likely to be touched when anyone shows her attention or respect, especially someone who claims to understand her. Thus, she might well express her joy and gratitude with exuberance. Nevertheless, Lu Lu’s response is, in our opinion, far from a “reasonable” overreaction. She deifies Cheng Yuanqing as a personal savior god who sees into the minds of others. This scene also exposes the reader to the potent and mysterious image of the psychotherapist for the Chinese public; for Lu Lu, what Cheng Yuanqing learned in the United States allows her to “detect the secrets of others.”

Exaggerations can be found throughout *Saving Breasts*. The author attributes the psychotherapist’s success to her “superpower” on the one hand and to the “magical” function of the therapy group on the other. The novel is inundated with optimism, which falsely suggests that therapy is a simple curative strategy mainly concerned with uncovering personal secrets. Group members are “healed” once they open up to each other about the origin of their psychological problems. Therapy is thereby simplified and reduced to a process of abreaction. The psychotherapist rarely interprets; when she speaks, we feel that she is providing didactic lessons (although this does not prevent her from enjoying the group’s admiration).

Cheng Yuanqing, who obtained her doctorate abroad, is described as a competent psychotherapist, while the protagonist of *The Woman Psychotherapist*, published 5 years later, is portrayed as having various difficulties in doing her job effectively. He Dun 贺顿, a fatherless girl whose mother remarried, leaves her village to earn for living. After a difficult period, she earns certification as a psychological counselor and opens her own consulting practice. Her practice runs smoothly until a patient’s husband informs her that his wife has attempted suicide. Disturbed, He Dun calls a famous psychologist for supervision, which leads He Dun to rediscover a traumatic event in her childhood and to understand her own behavior in response to it. Toward the end of the novel, feeling that she needs to develop her knowledge of psychology further, He Dun closes her practice to attend a 2-year training course.

Compared to *Saving Breasts*, the depiction of consultation in *The Woman Psychotherapist* is far more pertinent. Therapy is no longer represented in a simplistic way; the cases described are more complex, and analysis of each case is more developed. In the later novel, the author focuses on the difficulties He Dun encounters during her work. She realizes that the reason she is unable to respond to her clinical cases successfully is that they activate her own psychological issues. Thus, the process of overcoming her psychological complications also represents the process of her own maturation.

We believe that the significant differences between the two figures of the psychotherapist and how they are portrayed can be explained by Bi Shumin’s own clinical practice. *Saving Breasts* was published in 2003, the year when Bi Shumin founded her psychology practice. We assume that the novel was written before she had clinical experience and that the cases reported came from her imagination. *The Woman Psychotherapist* was published in 2008, by which time Bi Shumin had already acquired clinical experience as a psychotherapist, which is likely to have made her realize that therapy is not as simple as she once imagined.

The character of He Dun is representative of Chinese psychological counselors who ran private consulting practices at that time. Lacking a university education in psychology, He Dun nevertheless manages to practice the profession after obtaining a Psychological Counselor Certificate (*xinli zixunshi zigezheng* 心理咨询师资格证). In 2002, the Ministry of Labor and Social Security began issuing this certificate, which is obtained by completing 500 h of training and passing examinations. This was due to a reform in the mental health system initiated by the Chinese government, which “after more than two decades of lukewarm action, […] began to pay serious attention to mental health” (Huang and Kirsner, 2020, p. 8). The introduction of this policy significantly promoted the private practice of psychotherapy, which until that time had been rare and had existed mainly in public institutions such as hospitals and schools. The certificate’s introduction was a major factor in the emergence of the psychology boom.

The proliferation of psychological counselors running private practices also resulted from the industry’s lack of standardization and professionalization. The ease of acquiring a certificate and securing a job as a psychological counselor makes the profession a “low-investment, high-return, low-economic-risk” opportunity (Xia and Yang, 2007, p. 53), as mentioned in the novel by Tang Xiaoxi 汤小希, a counselor working at He Dun’s practice:

When it comes to psychotherapy, there are no professional standards. This business depends just on moral conscience. If we do our best, it is already good enough. […] On the other hand, the price–quality ratio of this work is really high. We earn money just by talking. Neither the wind nor the rain can touch us. Isn’t it a purely profitable business with no production costs at all! If the therapy has a curative effect, the patients are grateful. They see us as living Buddhas. If not, it’s because they don’t make an effort; they don’t wake up. Too bad for them, but it is not necessarily related to our work. Merchandise has guarantees or can even be returned. Doctors risk being sent to court if they’ve made a mistake or prescribed bad medication. But psychological counselors are well protected. The risk is almost zero (Bi, 2012, p. 439).

Tang points out that the biggest problem facing the psychotherapy profession in China is the lack of professional standards. Indeed, initially, the minimum educational requirements for a Level Three Psychological Counselor Certificate were a vocational high school (*zhongzhuan* 中专) diploma, which was replaced in 2005 by a college diploma, with no specification of major. However, due to “collusion between training agencies and local governments” (Huang, 2014, p. 190), many people who did not fulfill the conditions could pass the examinations to obtain the certificate. Thus, counselors trained through such short-term programs have become the subject of skepticism aimed not only at their professional skills but also at moral issues (see Huang, 2018, pp. 381–382). In the novel, Tang Xiaoxi, like He Dun, did not attend university. Her speech shows that her motivations are pecuniary and the health of her patients is unimportant. She is aware of the sector’s lack of regulation and exploits the psychic states of her patients to make money. In this context, the author discusses the subject of ethics in this emerging profession, which deserves special attention.

There is, however, an even more important problem highlighted in the novel: the incompetence of practitioners. He Dun finds the work very challenging, as she has not been trained systematically, undergone training analysis, or been guided by a supervisor. By means of the plot in which He Dun turns to a supervisor for help, Bi Shumin highlights the importance of supervisory support and personal analysis, which the psychoanalytic approach considers essential to therapists.

We would like to clarify that the therapies in the novel are psychoanalytic-oriented. The psychotherapists often establish connections between their patients’ psychological problems and past traumatic experiences, especially those that occurred during the patients’ childhood. Moreover, the name of the practice, 佛德, is suggestive. In choosing this name, He Dun is referring to Freud’s name translated into Chinese: 弗洛伊德. This corresponds to the real position of psychotherapy at that time in China. As Huang Hsuan-Ying remarked, when he was beginning his fieldwork in 2008, psychoanalysis “nearly became a generic term for the entire world of psychotherapy” (Huang, 2015, p. 16). Huang attributes this singular phenomenon to the applied training offered since 1997 by the German-Chinese Academy for Psychotherapy (德中心理治疗研究所) to foster the development of future counselors. These counselors were active in areas of clinical practice and training, eventually setting a basic framework in China for the counseling profession (especially for private practice). Psychoanalysis constitutes the core of these training sessions, which are delivered by German psychoanalysts and psychologists or psychiatrists. As Philippe Porret notes, “in the minds of both Chinese [trainees] and the [German] trainers, the term ‘psychotherapy’ designates a work of speech, using the manifestations of the unconscious, and thus requiring a knowledge of Freud’s concepts, and also of post-Freudian psychoanalysis” (Porret, 2008, p. 210).

Regarding training and supervision, training analysis is the key step in a psychoanalyst’s training; according to Freud, “we can only gain access to the practice of analysis by knowing our own unconscious” (Laplanche and Pontalis, 1967–2002, p. 25). In 1922, at the Congress of the International Psychoanalytical Association, the requirement for training analysis of all analytic candidates was established (Laplanche and Pontalis, 1967–2002, p. 26). Supervisory or supervised analysis – a prerequisite of securing certification to practice – is defined as “psychoanalysis conducted by an analyst in training, who reports periodically to an experienced analyst who guides him or her in understanding, who directs the treatment and helps the analyst in training to become aware of his or her countertransference” (Laplanche and Pontalis, 1967–2002, p. 353). Nevertheless, because psychotherapy started relatively recently in China, few experienced therapists were capable of doing training analysis or supervisory analysis at that time. It was not until the 2010s that Chinese therapists began to engage in regular supervision and personal analysis (Huang and Kirsner, 2020). Indeed, over the past decade or so, psychology, especially psychoanalysis, has grown rapidly in China. Several foreigner-taught programs, such as the Sino-German Course, the Sino-Norwegian Course, the Sino-American Course, and programs organized by the China American Psychoanalytic Alliance, have trained a significant number of therapists.

Through *The Woman Psychotherapist*, Bi Shumin highlighted the crucial problems prevalent in the psychology profession in China. The novel is particularly significant as it draws public attention to the field of work of psychotherapists, which presents certain risks for patients. By exposing details of consultations, which are generally aimed at revealing the impact of childhood trauma on personal development, the author draws attention to the mechanism of the psyche, and the novel as a whole can make the study of psychology more accessible to readers. Indeed, according to readers’ comments on Douban 豆瓣,^2^ many people bought the novel because of an interest in psychology.

As stated above, compared to *Saving Breasts*, consultations in *The Woman Psychotherapist* are described in a more authentic fashion. However, this does not mean that they are a flawless representation of a psychotherapist’s practice – far from it. With the exception of the complicated case that constitutes the main plot and being treated following the psychoanalytical approach, the other clinical cases presented in the novel appear simplistic. The author’s representation of the nuances of therapy is frequently reductive, and effective results are portrayed as being achieved with little therapeutic work. As Zhou Xue remarks, Bi Shumin relies substantially on mainstream values in consultations and guiding practices to achieve “happy ending[s],” and so fails to unearth the essence of psychological problems (Zhou, 2007, p. 170).

## Conclusion

We reviewed the figure of the psychotherapist in four literary works from contemporary China. Although fictional, these works reflect to some extent the evolution of psychology in China. We first noted the significant publication dates of these novels. The first two (by Xu Xiaobin and Ke Yunlu) were published in 1985 and 1987, respectively, corresponding to the period of the second Freudian fever in China. In contrast, the later novels of Bi Shumin, published in 2003 and 2008, are set in the context of the psychology boom that started from the early 2000s. However, it should be noted that these years represent the preliminary stage of the “psycho-boom,” as many in the field consider 2008-the year of the Wenchuan earthquake and the state’s promotion of psychological crisis intervention in its aftermath – as the “first year of psychology” (*xinli yuannian* 心理元年) (Huang, 2018, p. 376). After this period, several other novels were published, mainly by practitioners, such as Li Mengchao’s 李孟潮 *Interpretation of Obliteration* (*Mosha de jiexi* 抹杀的解析, 2010), Rong Weiling’s 荣伟玲 *Dream Interpretation Life* (*Shimeng rensheng* 释梦人生, 2018), Liu Zhao’s 刘昭 *Reflections on the Pupils* (*Yingtong* 映瞳, 2022), and Mei Mo’s 梅墨 *Psychological Counselor* (*Xinli zixunshi* 心理咨询师, 2022). These novels, whether published recently or some years ago, have not attracted much attention, probably due to the fact that the authors are not well known and that the works have been read by a relatively small audience. We thus excluded them from this study, since we focus on works written by famous writers and that are more widely known (although we might return to them in future research). We did not include any psychotherapist character in works published during the 1990s because the only relevant text published during this period that we were able to find was *The Psychotherapist is Here?* (*Xinli yisheng zaima?* 心理医生在吗? 1998) by Yan Geling 严歌苓, who, at the time of publication, had already lived in the United States for several years. Apart from psychoanalysis, this novel appears to have been influenced by the American psychologist Carl Rogers, particularly his work on client-centered therapy, and therefore does not fit with the development of psychotherapy in China. Indeed, the 1990s were marked by the cooling of the Freudian fever.

Regarding Xu Xiaobin’s and Ke Yunlu’s texts, in some ways, it is astonishing to see how specific basic concepts in psychoanalysis have been misinterpreted. However, the matter is also understandable given that the works were composed during the reintroduction of psychoanalysis in China. At that time, people in China only had access to a small portion of Freud’s theory. It was nearly impossible to learn how to apply these concepts in clinical practice. The figures of the psychotherapists in these works are therefore the products of pure imagination. The two psychotherapists composed by Bi Shumin, conversely, show a development in the understanding of psychoanalysis in China compared to its reception 20 years previously. Bi Shumin’s novels, which were published 5 years apart, show an evolution in the author’s understanding of a psychotherapist’s work. In *The Woman Psychotherapist*, the author, who had been practicing the profession for several years, reveals existing problems in the profession with a measure of verisimilitude and conveys a knowledge of psychology to the public. At the end of 2021, a television series based on *The Woman Psychotherapist* and bearing the same title was released. The timing of this adaptation, which appeared 13 years after the novel’s publication, was not arbitrary. The number of people experiencing mental health issues is rising; a nationwide survey published in 2019 estimated that 16.6 percent of the population had experienced mental disorders (Huang et al., 2019).^3^ Although mental health issues are increasing, there are a limited number of psychotherapists, and so there is a growing interest in mental health as a subject of public discourse. As a consequence, we expect that more figures of the psychotherapist will appear in contemporary Chinese literature to complement this topic of interest.

## Author Contributions

LG and WY contributed to the conception and design of the study. LG identified the literary materials, set the theoretical framework, and wrote the first draft of the manuscript. WY produced the second draft of the manuscript. Both authors contributed to manuscript revision and read and approved the submitted version.

## Conflict of Interest

The authors declare that the research was conducted in the absence of any commercial or financial relationships that could be construed as a potential conflict of interest.

## Publisher’s Note

All claims expressed in this article are solely those of the authors and do not necessarily represent those of their affiliated organizations, or those of the publisher, the editors and the reviewers. Any product that may be evaluated in this article, or claim that may be made by its manufacturer, is not guaranteed or endorsed by the publisher.

## Footnotes

1 All translations from other languages (French and Chinese) into English are made by the authors of this article, unless stated otherwise.

2 A website where people can comment on books, movies, or music albums (the most famous website of this kind in China).

3 The statistic concerns “weighted lifetime prevalence of any disorder excluding dementia.”
